# Obesity and cathepsin S in periodontal health and disease: A prospective clinical observational study

**DOI:** 10.2340/aos.v84.45208

**Published:** 2025-12-29

**Authors:** Ali Batuhan Bayırlı, Mehmetcan Uytun, İsmail Kırlı, Fulden Cantaş Türkiş, Ercan Saruhan, H. Gencay Keceli

**Affiliations:** a Department of Periodontology, School of Dentistry, Mugla Sıtkı Kocman University, Mugla, Türkiye; b Department of Internal Medicine, School of Medicine, Mugla Sıtkı Kocman University, Mugla, Türkiye; c Department of Basic Medical Sciences, School of Medicine, Mugla Sıtkı Kocman University, Mugla, Türkiye; d Department of Biochemistry, School of Medicine, Mugla Sıtkı Kocman University, Mugla, Türkiye; e Department of Periodontology, Faculty of Dentistry, Hacettepe University, Ankara, Türkiye

**Keywords:** Cathepsin S, obesity, periodontitis, nonsurgical periodontal therapy, saliva, gingival crevicular fluid

## Abstract

**Objectives:**

This study aimed to evaluate the association between obesity, periodontal status, and cathepsin S (CatS) levels in gingival crevicular fluid (GCF) and saliva and assess the impact of obesity on clinical and biochemical outcomes following nonsurgical periodontal therapy (NSPT).

**Methods:**

A total of 52 participants were categorized into nonobese with periodontal health, obese with periodontal health, nonobese with periodontitis, and obese with periodontitis groups. Clinical, periodontal, and anthropometric measurements were recorded. CatS levels in GCF and saliva were quantified using an enzyme-linked immunosorbent assay. NSPT was performed in the periodontitis groups, and clinical and biochemical parameters were re-evaluated after 3 months.

**Results:**

GCF and salivary CatS levels were highest in the obese periodontitis group and lowest in the nonobese periodontal health group (*p* < 0.001). Regression analysis revealed a significant positive association between body mass index (BMI), GCF, salivary CatS levels, and plaque index (*p* < 0.05). Significant positive correlations were observed between BMI and CatS levels, as well as between CatS levels and clinical periodontal parameters (*p* < 0.001). Following NSPT, both periodontitis groups exhibited significant clinical and biochemical improvement (*p* < 0.05). However, reductions in bleeding on probing, probing pocket depth, clinical attachment loss, and salivary CatS levels were significantly greater in the nonobese periodontitis group than in the obese group (*p* < 0.001).

**Conclusion:**

Periodontitis and obesity are associated with elevated CatS levels in GCF and saliva. Obesity may negatively impact clinical responses to NSPT. CatS could serve as a potential biomarker linking obesity to periodontitis and NSPT outcomes.

## Introduction

Maintaining oral health is recognized as a fundamental aspect of general health. The periodontal tissues function as a unified system to support the teeth [1]. The World Health Organization (WHO) describes health as more than simply the absence of disease, recognizing it as a holistic state that reflects physical health, mental stability, and social harmony [2]. In this context, periodontal health can be described as the absence of inflammatory periodontal conditions, enabling individuals to sustain their high quality of life [3]. Periodontitis is widely recognized as a chronic inflammatory condition driven by a dysbiotic shift in the oral microbiome, ultimately leading to the gradual breakdown of the periodontal tissues. Its main clinical manifestations typically present as gingival bleeding, clinical attachment loss (CAL), and periodontal pocket formation [4]. Owing to its high prevalence, periodontitis is a significant public health concern, contributing to tooth loss, diminished quality of life, and adverse systemic health outcomes [5]. Although microbial dental plaque constitutes the main etiological factor for the onset and progression of this condition, several systemic factors and enzymes are also involved in the shift from periodontal health to disease [6]. Among these, obesity and the cathepsin S (CatS) enzyme have drawn particular attention [7, 8].

Obesity is defined as an excessive accumulation of adipose tissue that negatively impacts overall health. This disease is a chronic, progressive, and recurrent condition with detrimental effects on both metabolic and psychosocial well-being [9–11]. It is commonly assessed using the body mass index (BMI) [12–14]. Obesity represents a significant risk factor for numerous chronic disorders, including cardiovascular diseases and diabetes [15, 16]. Several studies have established an association between obesity and oral diseases, particularly periodontitis [17–19]. Furthermore, the global prevalence of obesity is projected to increase substantially over the next decade [20].

CatS is an isoenzyme found in macrophages, human B-lymphocytes, and dendritic cells. It is an active cysteine protease involved in the degradation of essential extracellular matrix (ECM) components, including elastin and collagens, during immune responses [21–24]. Owing to its role in ECM degradation and involvement in inflammatory processes, CatS is considered to play a role in the development of periodontitis [25]. It contributes to collagen breakdown, osteoclast activation, and cytokine release, leading to connective tissue destruction and alveolar bone loss characteristic of periodontal disease [7, 25–27]. Recent studies have confirmed elevated CatS levels in inflamed periodontal tissues, further supporting its involvement in disease pathogenesis [26–28]. Transcriptomic analyses have shown that CatS is expressed in white adipose tissue and regulated by cytokines in human subcutaneous adipose tissue (scWAT). Moreover, obesity has been associated with CatS overexpression in scWAT and elevated circulating levels of this enzyme [29, 30].

Owing to the challenges in standardizing the assessment of periodontal and systemic conditions among clinicians, combined with the potential effects of individual patient factors, there is a growing need for more objective diagnostic tools. Salivary and gingival crevicular fluid (GCF) biomarkers provide a noninvasive and objective alternative for evaluating periodontal status [31–33]. These biomarkers have been linked to periodontitis-related changes, including inflammation, ECM degradation, and alveolar bone turnover [33–35]. Therefore, GCF and saliva were selected as sample types to assess CatS levels in our study.

Previous studies have demonstrated that CatS is involved in periodontal inflammation and tissue degradation [7, 25]. However, the potential synergistic effect of obesity, an established pro-inflammatory condition, on CatS expression within the periodontium has not been fully elucidated. While both periodontitis and obesity are characterised by chronic low-grade inflammation, limited evidence exists regarding how obesity-related systemic inflammation influences local CatS activity in GCF and saliva. Therefore, this study aimed to address the current knowledge gap by elucidating the associations among obesity, GCF, and salivary CatS levels in individuals with periodontal health and periodontitis. Moreover, it sought to determine the influence of obesity on the clinical and biochemical responses to nonsurgical periodontal therapy (NSPT), thereby providing new insights into the mechanistic link between systemic metabolic inflammation and local periodontal condition.

## Methods

### Study design and participants

This study was approved by the Medical Sciences Ethics Committee of Mugla Sıtkı Kocman University (Approval No: 146) on November 6, 2024 and conducted in accordance with the principles of the Declaration of Helsinki. All participants were thoroughly informed about the study’s purpose and procedures, and written informed consent was obtained. The study was conducted and reported following the CONSORT guidelines. Clinical and radiographic examinations were performed at the Department of Periodontology, Faculty of Dentistry, Mugla Sıtkı Kocman University. A total of 52 individuals meeting the following criteria were included in the study: (1) age between 25 and 40 years, which was selected to minimize age-related variability in periodontal destruction and systemic comorbidities. Although this represents a relatively young range for severe periodontitis, it allows for evaluating the influence of obesity on periodontal and biochemical parameters in a metabolically active and homogeneous population [36–38], (2) nonsmokers, (3) absence of systemic diseases other than obesity, (4) no regular use of systemic or topical medication, and (5) no history of NSPT within the past 3 months. Exclusion criteria were: (1) presence of systemic diseases other than obesity, (2) current smoking, (3) pregnancy or lactation, (4) use of systemic or topical medication within the previous 3 months, (5) history of NSPT within the past 3 months, and (6) fewer than 20 teeth present. No participants were excluded after the eligibility assessment.

### Study groups

This study was designed as a prospective clinical observational study. Fifty-two participants were classified into four groups according to their periodontal status, as either periodontally healthy or having periodontitis, and their BMI category, as either normal weight or obese. Classification was based on clinical and anthropometric measurements. In November 2024, volunteers were first evaluated for obesity criteria at the Department of Internal Medicine, Faculty of Medicine, Mugla Sıtkı Kocman University before being referred to the Department of Periodontology, Faculty of Dentistry. Obesity was classified according to the cutoff values defined by the WHO: individuals with a BMI of ≥30 kg/m² were categorized as obese, while those with a BMI between 18.5 and 24.9 kg/m² were classified as normal weight [39]. Individuals with intermediate BMI values (25.0–29.9 kg/m²) or with gingivitis were excluded during screening to ensure clear group differentiation. Periodontal examinations assessed the following: plaque index (PI), gingival index (GI), bleeding on probing (BOP), probing pocket depth (PPD), and CAL. Periodontal diagnoses of the participants in each group were determined according to the 2017 classification system established by the European Federation of Periodontology and the American Academy of Periodontology [3, 4]. The study groups and their inclusion criteria were as follows:

Group I (nonobese periodontally healthy) (*n* = 13): CAL ≤ 1 mm and PPD ≤ 3 mm on all teeth, no radiographic bone loss, BOP < 10%, and a BMI between 18.5 and 24.9 kg/m².

Group II (obese periodontally healthy) (*n* = 13): CAL ≤ 1 mm and PPD ≤ 3 mm on all teeth, no radiographic bone loss, BOP < 10%, and BMI ≥ 30 kg/m².

Group III (nonobese periodontitis) (*n* = 13): Loss of no more than four teeth owing to periodontal reasons, CAL ≥ 5 mm and PPD ≥ 6 mm on at least one tooth, radiographic bone loss extending to the middle or apical third of the root (stage III grade B periodontitis), and BMI between 18.5 and 24.9 kg/m².

Group IV (obese periodontitis) (*n* = 13): Loss of no more than four teeth owing to periodontal reasons, CAL ≥ 5 mm and PPD ≥ 6 mm on at least one tooth, radiographic bone loss extending to the middle or apical third of the root (stage III grade B periodontitis), and BMI ≥ 30 kg/m².

NSPT protocol: NSPT was administered to patients with periodontitis three times at 1-month intervals. The therapy consisted of full-mouth supra- and subgingival scaling and root planing performed under local anesthesia as required, using ultrasonic scalers (Cavitron Select SPS, Dentsply Sirona, USA) and Gracey curettes (Hu-Friedy, Chicago, IL, USA). All procedures were performed by a single calibrated periodontist to ensure standardization. No systemic or local antimicrobials or chemical adjuncts were prescribed. Participants received standardized oral-hygiene instructions and professional plaque control at each visit. Re-evaluation was conducted 3 months after baseline.

### Periodontal examination

All participants underwent clinical and radiographic examinations of the periodontal tissues. Clinical measurements were recorded at six sites per tooth using a Williams periodontal probe (Hu-Friedy Manufacturing Co. Inc., Chicago, IL, USA). Examinations were conducted by two periodontologists (A.B.B. and M.U.). Prior to the study, calibration was performed on 15 patients with stage III periodontitis. Intra- and inter-examiner reliabilities were assessed using the intraclass correlation coefficient (ICC). Intra-examiner ICC values were 0.92 (PPD) and 0.91 (CAL) for A.B.B. and 0.90 (PPD) and 0.88 (CAL) for M.U. Inter-examiner ICC values were calculated as 0.90 (PPD) and 0.91 (CAL). Based on these results, the measurements were considered reliable.

### Saliva sample acquisition

Unstimulated saliva samples were obtained using the passive drool technique [40]. A single sample was obtained from individuals in the periodontally healthy groups, whereas samples were obtained twice from the periodontitis groups, before and 3 months after treatment. To standardize the procedure and minimize biological variation, all samples were collected between 8:00 and 10:00 a.m. after an overnight fast in Eppendorf tubes. Female participants were scheduled outside the menstrual phase of their cycle to minimize potential hormonal influences on biomarker levels. Before providing 2–4 mL of saliva, participants were instructed to rinse their mouths with water for 2 min to remove food debris. The collected samples were centrifuged at 1,000 × g for 10 min, and the supernatants were transferred into new Eppendorf tubes and stored at −80°C until biochemical analysis.

### GCF sample acquisition

In the periodontal health groups, GCF samples were obtained from the vestibular surfaces of the maxillary central incisors. A single sample was obtained from each individual in the healthy group, while samples were obtained twice from patients with periodontitis before treatment and at 3-month follow-up. In the healthy group, all teeth were present, with no tooth loss. For the periodontitis groups, sampling sites were selected from teeth with the deepest periodontal pockets (PPD ≥ 6 mm). Each patient provided a sample from one site on a single tooth. Prior to the collection of GCF samples, supragingival plaque was eliminated using a sterile scaler (Hu-Friedy Manufacturing Co. Inc., Chicago, IL, USA) without contacting the gingival margin. The tooth was isolated using a cotton roll and saliva ejector and before being dried with a sterile 2 × 2 gauze. A paper strip was used at each site. GCF samples were collected using standardized 2 × 14 mm paper strips (Periopaper^®^, Pro Flow Inc., Amityville, NY, USA) designed for optimal absorption. These strips were inserted into the gingival sulcus and left in place for 30 s. To ensure procedural consistency and minimize biological variability, all GCF samples were collected between 8:00 and 10:00 a.m. after an overnight fast. Female participants were scheduled outside the menstrual phase of their cycle to avoid hormonal influences on GCF biomarker levels. Any strips contaminated with saliva or blood were excluded from further evaluation. The collected strips were placed in Eppendorf tubes containing 300 μL of phosphate-buffered saline and centrifuged at 1,000 × g for 10 min. The resulting supernatants were separated for analysis and stored at −80°C until the day of the experiment.

### Cathepsin S measurements

GCF and salivary CatS levels were determined by means of a human CatS enzyme-linked immunosorbent assay (ELISA) kit (Cat# E0712Hu, BT-Laboratory, Shanghai, China) following the manufacturer’s instructions. The measurements were obtained with the aid of an ELISA plate reader (Multiskan GO microplate reader, Thermo Fisher Scientific, Waltham, MA, USA). The assay sensitivity was 14.51 ng/L with inter- and intra-assay coefficients of variation of < 10%.

### Anthropometric measurements

All participants underwent anthropometric measurements following standardized protocols. In the periodontitis groups, measurements were repeated after treatment to confirm the diagnosis of obesity and nonobesity. All measurements were performed by a single internal medicine specialist (İ.K.) using the same calibrated instrument to minimize inter-measurement variability and ensure compliance with international standards. Each measurement was performed twice, and the mean value was used for analysis. The following parameters were assessed [12–14]:

BMI: Body weight and height were measured using a calibrated digital scale while participants wore light clothing and no shoes. BMI was calculated using the formula: body mass (kg)/height² (m²). Individuals with a BMI ≥ 30 kg/m² were classified as obese, while those with a BMI between 18.5 and 24.9 kg/m² were classified as nonobese.

Waist circumference (WC): Measurements were taken while participants stood upright after normal exhalation, with relaxed abdominal muscles. A nonstretchable measuring tape was used at the midpoint between the lower costal margin and the iliac crest. A WC of ≥ 90 cm in women and ≥ 100 cm in men was considered indicative of central obesity.

Waist-to-height ratio (WHtR): Calculated by dividing waist circumference (cm) by height (cm). A WHtR ≥ 0.5 was considered the cut-off value for central obesity.

Waist-to-hip ratio (WHR): Calculated by dividing waist circumference (cm) by hip circumference (cm). The hip circumference was measured at the widest part of the gluteal region while participants stood upright. A WHR ≥ 0.85 in women and ≥ 0.9 in men was considered the cut-off value for central obesity.

### Statistical analysis

All statistical analyses were conducted using SPSS version 27.0 (IBM Corp., Armonk, NY, USA) and R version 2024.09.0 (R Foundation for Statistical Computing, Vienna, Austria). Continuous variables were presented as mean ± standard deviation or median (minimum–maximum), depending on the distribution characteristics, while categorical variables were expressed as frequencies and percentages (%). The normality of data distribution was assessed using the Kolmogorov–Smirnov test. Comparisons of continuous variables among groups were conducted using one-way analysis of variance (ANOVA) for normally distributed data, followed by Tukey’s or Tamhane’s T2 post-hoc test depending on the homogeneity of variances. For non-normally distributed data, the Kruskal–Wallis test was applied, followed by Bonferroni-corrected Mann–Whitney U tests for pairwise comparisons. For dependent (paired) measurements, the paired t-test was used when normality assumptions were met; otherwise, the Wilcoxon signed-rank test was performed. To evaluate the interaction between time (baseline vs. third month) and group (obese periodontitis vs. nonobese periodontitis), two-way repeated measures ANOVA was conducted, and Bonferroni post-hoc tests were used for multiple comparisons. Differences between categorical variables were analyzed using the chi-squared test. Correlations between continuous variables were assessed using Pearson’s correlation coefficient, and the correlation matrix was visualized as a heat map to facilitate interpretation. In addition, simple linear regression analysis was conducted to examine the linear relationships between the selected variables. A *p*-value of <0.05 was considered statistically significant for all analyses. To calculate the sample size, data obtained from the study by Türkoğlu et al. [41] were used, and power analyses were performed using the G Power statistical software. The calculation indicated that a total of 44 participants would be sufficient to detect a medium effect size (*f* = 0.25) with 95% power and a significance level of 0.05. To account for potential dropouts, the total number of participants was increased to 52.

## Results

In our study, clinical parameters were analyzed alongside GCF and saliva samples collected from 52 individuals. There were no statistically significant differences in terms of sex distribution (*p* = 0.999) or mean age (*p* = 0.978) among the four groups. Anthropometric measurements showed the highest values in the obese groups, which were significantly different from those in the nonobese groups (*p* < 0.001). All clinical periodontal parameters were higher in the periodontitis groups than in the healthy groups (*p* < 0.001). In addition, PI values were significantly higher in the obese periodontitis group than in the nonobese periodontitis group (*p* < 0.001). Biochemical analysis showed that both GCF and salivary CatS levels were significantly elevated in the obese periodontitis group and lowest in the nonobese periodontal healthy group (*p* < 0.001) (Table 1).

**Table 1:** Clinical, biochemical, and demographic data.

Variables (baseline)	Nonobese periodontal health (I) (*n* = 13)	Obese periodontal health (II) (*n* = 13)	Nonobese periodontitis (III) (*n* = 13)	Obese periodontitis (IV) *n* = 13)	F / *_Χ_*^2^	*p*-value
**Sex**					0.000	> 0.999
Male	7 (53.8)	7 (53.8)	7 (53.8)	7 (53.8)		
Female	6 (46.2)	6 (46.2)	6 (46.2)	6 (46.2)		
Age (years)	31 ± 2.58	31.15 ± 2.15	30.92 ± 2.63	30.77 ± 1.48	0.065	0.978
BMI (kg/m^2^)	21.16 ± 0.98	32.71 ± 1.20	21.12 ± 1.07	32.85 ± 1.26	457.056	**< 0.001^a,c,d,f^**
WC (cm)	80 (66–92)	103 (92.50–108)	78 (66–90)	102 (91–115)	37.575	**< 0.001^a,c,d,f^**
WHR	0.77 (0.72–0.83)	1.11 (0.90–1.15)	0.74 (0.67–0.88)	1.11 (0.87–1.21)	38.081	**< 0.001^a,c,d,f^**
WHtR	0.45 (0.41–0.48)	0.60 (0.53–0.69)	0.43 (0.41–0.48)	0.61 (0.58–0.67)	39.486	**< 0.001^a,c,d,f^**
GCF CatS (ng/L)	574.54 ± 136.62	915.92 ± 107.86	2552.46 ± 310.81	4297.46 ± 462.48	445.652	**< 0.001^g^**
Saliva CatS (ng/L)	654.46 ± 125.73	960.31 ± 99.29	2868.85 ± 283.66	4350.69 ± 587.07	345.990	**< 0.001^g^**
PI	0.90 (0.66–1.09)	1.09 (0.71–1.27)	1.47 (0.74–2.38)	2.37 (1.36–2.68)	34.272	**< 0.001^b,c,e,f^**
GI	0.98 (0.74–1.13)	0.96 (0.73–1.13)	1.67 (1.09–2.32)	2.30 (1.68–2.38)	38.291	**< 0.001^b,c,d,e^**
BOP (%)	8.63 ± 0.96	8.99 ± 0.88	46.02 ± 23.17	64.70 ± 12.66	58.046	**< 0.001^b,c,d,e^**
PPD (mm)	1.79 ± 0.27	1.90 ± 0.35	3.52 ± 0.52	3.92 ± 0.38	102.648	**< 0.001^b,c,d,e^**
CAL (mm)	0.62 (0.38–0.91)	0.68 (0.46–0.91)	3.47 (1.95–4.88)	3.50 (3.22–4.88)	39.214	**< 0.001^b,c,d,e^**

Note: Data are represented as mean ± SD or median (min.-max.). Bold values indicate statistical significance (*p* < 0.001). Different superscript letters indicate statistically significant differences between groups based on post-hoc analysis (*p* < 0.01, all pairwise comparisons). F / *χ*² indicates test statistics.

Abbreviations: BMI: body mass index; WC: waist circumference; WHR: waist-to-hip ratio; WHtR: waist-to-height ratio; CatS: Cathepsin S; CAL: clinical attachment loss; GCF: gingival crevicular fluid; BOP: bleeding on probing; GI: gingival index; PPD: probing pocket depth; PI: plaque index; *n*: number of samples.

a Statistically significant difference between groups I and II.

b Statistically significant difference between groups I and III.

c Statistically significant difference between groups I and IV.

d Statistically significant difference between groups II and III.

e Statistically significant difference between groups II and IV.

f Statistically significant difference between groups III and IV.

g Statistically significant difference between all groups.

**Figure 1:**
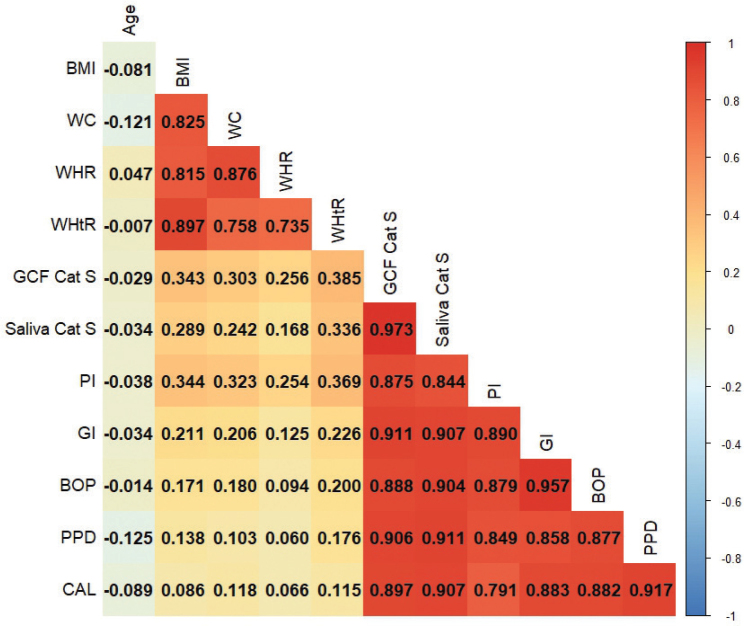
Correlation analysis among clinical, biochemical, and anthropometric variables. Note: Pearson’s correlation coefficients are visualized using a color gradient, with red indicating strong positive correlations and blue indicating strong negative correlations. Only statistically significant correlations (*p* < 0.05) were considered for interpretation. For clarity, only the lower triangle of the symmetric matrix is shown. Abbreviations: BMI: body mass index; WC: waist circumference; WHR: waist-to-hip ratio; WHtR: waist-to-height ratio; CatS: Cathepsin S; CAL, clinical attachment loss; GCF: gingival crevicular fluid; BOP: bleeding on probing; GI: gingival index; PPD: probing pocket depth; PI: plaque index.

The relationship between BMI and clinical and biochemical parameters at baseline was assessed using simple linear regression and Pearson’s correlation analyses. According to the regression analysis, BMI had a positive and statistically significant effect on GCF and salivary CatS levels, as well as on PI (*p* < 0.05). No significant effects were observed on other clinical periodontal parameters. Figure 1 illustrates the correlation matrix displaying the interrelationships among anthropometric indices, GCF and salivary CatS, and clinical periodontal parameters. These findings were supported by the correlation analysis, which revealed significant positive correlations between BMI and CatS levels (*p* < 0.001). Additionally, strong and significant positive correlations were identified between GCF and salivary CatS levels and clinical periodontal parameters (*p* < 0.001). A particularly strong positive correlation was observed between GCF and salivary CatS levels, whereas the strongest anthropometric correlation was found between BMI and WHtR (*p* < 0.001; Figure 1).

**Figure 2:**
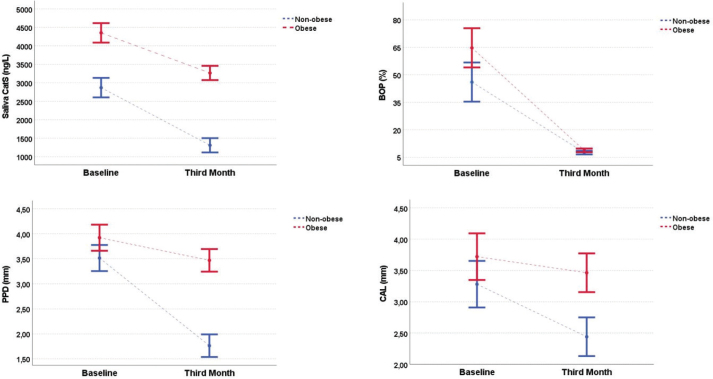
Interaction analyses and changes in clinical and biochemical parameters between nonobese and obese individuals with periodontitis from baseline to the third month following nonsurgical periodontal therapy. Note: The graphs show the changes in salivary CatS, BOP, PPD, and CAL levels from baseline to the third month after nonsurgical periodontal therapy. Values are represented as the means ± standard error. Two-way repeated-measures analysis of variance was used to assess the interaction effect between time and obesity status. Significant group × time interactions were observed for all parameters (*p* < 0.05), indicating greater improvement in the nonobese periodontitis group. Dashed blue lines represent nonobese participants with periodontitis, while dashed red lines represent obese periodontitis participants. Abbreviations: CatS: Cathepsin S; CAL: clinical attachment loss; BOP: bleeding on probing; PPD: probing pocket depth.

Table 2 presents the clinical, periodontal, and biochemical parameters before and after the NSPT in the periodontitis groups. In both groups, all periodontal parameter values demonstrated a statistically significant reduction post-treatment compared to baseline (*p* < 0.05). Specifically, mean PPD decreased from 3.52 ± 0.52 mm to 1.76 ± 0.29 mm in the nonobese periodontitis group and from 3.92 ± 0.38 mm to 3.47 ± 0.48 mm in the obese periodontitis group, corresponding to mean reductions of 1.76 mm and 0.45 mm, respectively. Similarly, CAL improved by approximately 0.9 mm and 0.3 mm in the nonobese and obese groups, respectively. Both groups showed statistically significant improvements (*p* < 0.001). Furthermore, GCF and salivary CatS levels significantly decreased following treatment in both groups (*p* < 0.001). Figure 2 illustrates the interaction effects between time and obesity status, showing the longitudinal changes in salivary CatS, BOP, PPD, and CAL from baseline to the third month after NSPT. When comparing the changes in clinical parameters between the two groups after NSPT, the reductions in BOP, PPD, and CAL were significantly greater in the nonobese periodontitis group than in the obese group (*p* < 0.001). In addition, the decrease in salivary CatS levels following NSPT was significantly greater in the nonobese periodontitis group (*p* < 0.001).

**Table 2:** Clinical and biochemical parameters at baseline and after periodontal treatment in periodontitis groups.

	Nonobese periodontitis (*n* = 13)	Obese periodontitis (*n* = 13)
GCF CatS		
Baseline	2552.46 ± 310.81	4297.46 ± 462.48
Within the group	**< 0.001**	**< 0.001**
Month 3	915.85 ± 71.42	2577.08 ± 250.05
Saliva CatS		
Baseline	2868.85 ± 283.66	4350.69 ± 587.07
Within the group	**< 0.001**	**< 0.001**
Month 3	1,309 ± 144.04	3265.38 ± 454.47
PI		
Baseline	1.56 ± 0.57	2.37 (1.36–2.68)
Within the group	**< 0.001**	**0.001**
Month 3	0.76 ± 0.17	1.36 (0.55–1.77)
GI		
Baseline	1.67 (1.09–2.32)	2.30 (1.68–2.38)
Within the group	**0.030**	**0.001**
Month 3	1.20 (0.86–1.96)	1.29 (1.06–1.80)
BOP		
Baseline	52.38 (16.60–80)	66 (43.75–80)
Within the group	**0.001**	**0.001**
Month 3	8.75 (4.72–9.86)	9.30 (5.83–9.90)
PPD		
Baseline	3.52 ± 0.52	3.92 ± 0.38
Within the group	**< 0.001**	**< 0.001**
Month 3	1.76 ± 0.29	3.47 ± 0.48
CAL		
Baseline	3.47 (1.95–4.88)	3.50 (3.22–4.88)
Within the group	**0.001**	**0.001**
Month 3	2.56 (1.17–3.47)	3.22 (3–4.35)

Note: Bold values indicate statistical significance (*p* < 0.05).

Abbreviations: CatS: Cathepsin S; CAL: clinical attachment loss; GCF: gingival crevicular fluid; BOP: bleeding on probing; GI: gingival index; PPD: probing pocket depth; PI: plaque index; *n*: number of samples.

## Discussion

The aim of this study was to investigate the effect of obesity on GCF and saliva levels of CatS and periodontal parameters in periodontal health and disease before and after NSPT. In line with previous studies, in both groups including obese individuals, clinical parameters demonstrating the severity of the periodontal inflammation and destruction showed a tendency to be higher compared to the nonobese individuals [42–44]. Moreover, CatS levels in both saliva and GCF were higher in individuals with obesity compared to individuals without obesity. Previous studies have also reported that CatS overexpression and elevated circulating levels are linked to obesity [29, 30]. Cytokines such as TNF-α and IL-6 released from adipose tissue can induce low-grade systemic inflammation, altering the host response to dental biofilm and thereby promoting periodontal destruction. The positive association between CatS and systemic inflammatory markers such as C-reactive protein and IL-6 observed in obese individuals supports this relationship [45]. Collectively, these findings, together with our results, may explain the elevated CatS levels detected in obesity. Additionally, CatS inhibitors have been shown to reduce inflammation by modulating macrophage activity and cytokine release, highlighting CatS as a potential therapeutic target in obesity-related inflammatory conditions [46].

In this study, linear regression analysis revealed a positive effect of BMI on baseline GCF and salivary CatS levels, indicating that higher BMI corresponds to increased CatS concentrations, consistent with previous findings. The highest GCF and salivary CatS levels were observed in the obese periodontitis group and the lowest in the nonobese healthy group, suggesting a cumulative effect of obesity and periodontitis on CatS elevation. This coexistence was also reflected in clinical parameters, as PI values were higher in obese individuals with periodontitis, and BMI had a positive effect on PI. In obese individuals, inadequate oral hygiene may contribute to greater plaque accumulation and disease severity [17, 47, 48]. Similarly, previous studies reported significantly higher PI, CAL, and PPD values in obese periodontitis patients compared with their nonobese counterparts [42, 43].

CatS has been implicated in bone loss during periodontitis progression [49]. Its expression increases in periodontal tissues under inflammatory and infectious conditions, suggesting an autophagy-related role in disease pathogenesis [25]. In an animal model, Gonzales et al. reported enhanced CatS gene expression in gingival biopsies, further linking CatS to periodontal tissue destruction [28]. These observations are consistent with our findings of elevated salivary and GCF CatS levels in patients with periodontitis compared to healthy individuals. CatS has been suggested to participate in immune regulation through its protease activity and involvement in antigen presentation pathways [26]. Experimental data indicate that CatS may also contribute to inflammatory signaling in response to periodontal pathogens [27]. Although the present study did not assess these mechanisms directly, previous evidence implies that CatS might be linked to oxidative and inflammatory processes that are relevant to both obesity and periodontitis [50–52].

CatS levels were significantly higher in patients with periodontitis and positively correlated with clinical periodontal parameters, suggesting that CatS expression increases in parallel with disease severity. The highest concentrations were observed in the obese periodontitis group, followed by the nonobese periodontitis, obese healthy, and nonobese healthy groups, indicating that periodontitis exerts a stronger influence on CatS levels than obesity. This dominant effect may be explained by the central role of CatS in periodontal-specific mechanisms such as biofilm-induced immune activation, lysosomal protease activity, antigen presentation via MHC class II, and Th17-mediated inflammation, rather than in obesity-related pathways like systemic low-grade inflammation or adipokine imbalance [26, 27]. Further studies evaluating the combined impact of obesity and periodontitis within the same cohort are warranted to confirm these observations.

Altay et al. [42] examined serum inflammatory parameters after NSPT in periodontitis with and without obesity and found significant clinical improvements in both groups, with greater reductions in CAL and systemic inflammatory markers in obese participants. Conversely, a previous study showed that obesity may predict a reduced response to NSPT [43], whereas another reported a more pronounced PPD reduction in obese individuals [44]. A systematic review and meta-analysis [53] concluded that periodontal treatment is generally effective irrespective of obesity, yet effects on inflammatory biomarkers remain uncertain. In our study, greater improvements in BOP, CAL, and PPD occurred in nonobese individuals, suggesting that obesity may attenuate periodontal healing via persistent systemic inflammation. These findings underscore the need for individualized periodontal management in obesity. These inconsistencies among studies may be attributed to variations in sample type, follow-up duration, baseline disease severity, and the systemic inflammatory status of obese individuals, all of which can differentially influence the clinical and biochemical response to NSPT.

In the present study, the analysis of NSPT outcomes showed that clinical periodontal parameters significantly improved following treatment in both periodontitis groups compared to baseline. Similarly, a marked reduction in the CatS levels was observed. Pearson’s correlation analysis based on baseline values demonstrated strong positive correlations between CatS concentration and clinical periodontal parameters. In addition, a significant and strong positive correlation was found between CatS levels in the GCF and saliva. These findings suggest that post-treatment reductions in both clinical and biochemical parameters are expected to the effectiveness of NSPT. On the other hand, NSPT significantly reduced CatS levels in both saliva and GCF of individuals with periodontitis. However, obesity limited the clinical and biochemical improvements achieved after NSPT and reduced the success of periodontal treatment as same as its influence on the occurrence of the disease.

To our knowledge, no previous study has simultaneously evaluated GCF and salivary CatS levels in relation to periodontal status, making direct comparisons difficult. In this study, salivary and GCF CatS concentrations were positively correlated, and both decreased after NSPT. A significantly greater reduction in salivary CatS was observed in nonobese individuals, whereas GCF CatS levels showed no intergroup difference. This indicates that systemic inflammation linked to obesity more strongly influences salivary CatS, while local periodontal healing appears comparable between groups. The parallel decline and correlation of CatS levels in both fluids suggest that saliva and GCF similarly reflect periodontal inflammation. These findings emphasize the diagnostic and prognostic potential of CatS as a noninvasive biomarker for monitoring periodontal disease. Although not yet included in current classification systems, biomarkers such as CatS may contribute to future personalized diagnostic and treatment approaches [54, 55].

One of the main strengths of this study is its combined cross-sectional and longitudinal design, which enabled the evaluation of both short- and long-term outcomes. Multiple anthropometric measures were used in addition to BMI, providing a more comprehensive assessment, and these were repeated at follow-up to ensure consistency of obesity status. However, several limitations should be acknowledged. The follow-up period was relatively short (3 months), and the sample size modest, limiting generalizability. Long-term studies with larger and more diverse cohorts are needed to confirm these findings. Genetic and lifestyle factors that may influence both obesity and periodontal disease were not assessed. Although sex distribution was balanced across the study groups, the inclusion of both female and male participants may have introduced hormonal variability that could influence inflammatory and biochemical parameters such as CatS levels. Moreover, circulating CatS levels may not directly reflect enzymatic activity, as CatS can be inactivated by binding to its endogenous inhibitor cystatin C [56]. Considering cystatin C levels is therefore important when interpreting CatS activity, especially in metabolic conditions such as obesity and atherosclerosis [57]. Despite these limitations, this study provides valuable insight into the relationship among obesity, CatS, and periodontal inflammation.

## Conclusion

This study demonstrated that obesity significantly elevated CatS levels in both GCF and saliva. Moreover, the positive correlation between CatS levels and clinical periodontal parameters, along with the post-treatment reduction in CatS concentration, supports the role of this enzyme in the progression of periodontal disease. In addition, obesity has been shown to negatively impact periodontal treatment outcomes. These findings suggest that the relationship between obesity and CatS levels may be clinically significant for the management of periodontal disease.

## Data Availability

The data supporting the findings of the present study are available from the corresponding author upon request.
